# The item position effects in international examinations: the roles of gender

**DOI:** 10.3389/fpsyg.2023.1220384

**Published:** 2023-08-15

**Authors:** Georgios Sideridis, Hailah Hamed, Fathima Jaffari

**Affiliations:** ^1^Boston Children’s Hospital, Harvard Medical School, Boston, MA, United States; ^2^Department of Primary Education, National and Kapodistrian University of Athens, Zografou, Greece; ^3^Education and Training Evaluation Commission, Riyadh, Saudi Arabia

**Keywords:** item position effects, national examinations, teacher licensure test, Mplus, MIMIC model

## Abstract

The goal of the present study was to evaluate the roles of item position in terms of item difficulty levels in the assessment of aptitude. Using data from a National Examination in Saudi Arabia, the item position effect was evaluated as a teacher licensure test (GTLT) was administered using five different forms with the same items appearing in a different order. Results indicated minuscule in magnitude position effects estimates, overall, with initially 11.1% of the tests being significant but all of them failing to reach significance using the Holm–Bonferroni’s and Sidak corrective procedures. With regard to gender, item position effects emerged in 47.6% of the tests after adjusting the level of significance using the Sidak correction. Interestingly, the direction of effect was consistent so that in 87% of the significant gender comparisons, item position effects were in the direction where females were spending more time on items when they appeared in later positions on the test compared to males. Assuming that items appearing later on the test are likely more difficult, the present findings suggest a profile of deep processing and active engagement in females when facing achievement tests.

## 1. Introduction

The sequence in which things are presented on a test can have an impact on a person’s performance on the test by influencing how they respond to later questions as a result of having seen earlier questions (Knowles, 1988). This is because the brain has a limited capacity for processing information, and when it is confronted with a great amount of information all at once, it may have difficulties keeping and organizing all of the information. As a consequence, people may have better results on exams when the questions are posed in a manner that is more rational and well-organized, which makes it simpler for the brain to comprehend the information. Also, a person’s performance on a test might be affected by the amount of difficulty of the items that are being tested on. If the questions are excessively challenging and they emerge early in the exam, they may produce frustration and a drop in performance; if the challenging questions appear later on the exam, participants may suffer from exhaustion, fatigue, and a loss in motivation as a result of being engaged with the exam for a relatively long time (Hambleton and Traub, 1974). Consequently, a person’s overall performance on a test can be significantly affected by factors such as the difficulty level of the questions, as well as the order in which they appear on the exam.

Item location effects refer to the phenomenon in which the placement and positioning of a test item within the exam may impact the examinee’s response to that item. Two types of effects have been identified, effects on item level or item’s variability (Albano, 2013; Weirich et al., 2017; Demirkol and Kelecioglu, 2022) both of which are detrimental for measurement purposes. In the present study we focus on the effects on item level rather than variability assuming that item position exerts systematic effects over all participants (i.e., being a source of systematic measurement error) rather than it varies between participants. This effect could affect the reliability of a test, as test-takers’ responses may vary depending on the location of a question (Ackerman et al., 2013; Streiner et al., 2016). If this occurs, test results may not be consistent over time or between various test versions (Anastasi and Urbina, 1997). Numerous studies have been conducted on the effects of item placement; for instance, Debeer and Janssen (2013) found substantial effects of item location on test performance in large-scale testing. Comparatively, to when the same items were placed earlier in the exam, they discovered that items situated near the end of the exam typically received lower scores (see also Haladyna and Downing, 2004; Debeer et al., 2014). In a similar vein, Lindner et al. (2015) observed that questions situated at the beginning of a test were more likely to be answered accurately. They attributed this result to a possible convergence of factors, such as increased attention, motivation, or a lack of fatigue at the start of the test. It is important to recognize that not all studies identify strong item location effects. According to the findings of Wise and DeMars (2006), item location effects may be negligible and significantly dependent on the exam’s particular characteristics (see also, Wise et al., 2004). The presence of item position effects poses a significant threat to the validity of test results (Lord and Novick, 1968) and the conclusions drawn from them (Zlatkin-Troitschanskaia et al., 2019), particularly to test fairness. If test-takers respond with greater accuracy to items at the beginning of the test (i.e., primacy effect) or with greater error to items at the end of the test (i.e., recency effect), then the test results will be biased toward those items (Murphy and Davidshofer, 2005; Paek, 2018). In other words, one of the most important characteristics of items, item difficulty, interacts with context, i.e., item position, to obfuscate measurement results (Zeller et al., 2017). This can cast doubt on claims of interchangeability between test forms. In addition, test-takers may not provide equally reliable responses to early and later questions because they may employ different responding strategies and levels of effort because their response to later questions depends on their prior experience, expectations, and perceptions of success. Rose et al. (2019) suggested that as a test progresses, a test-taker’s performance may decrease due to factors such as fatigue or loss of motivation (Baumeister and Vohs, 2007). The opposite can also be true; that is, as participants become better acquainted with the content or method of assessment their performance on later items can increase or change in variability (Whitely and Dawis, 1976).

### 1.2. Importance of the present study

The current study is significant because it analyzes a systematic source of measurement error associated with altered performance due to item position. This type of inaccuracy has the potential to render a person’s estimations of their skills and competencies inaccurate. For instance, questions that come later in a test may reflect responses that are a result of factors such as fatigue (Ackerman and Kanfer, 2009), motivation (Grant and Dweck, 2003) or a lack of it, interest (Butler, 2006), primacy effects, anxiety, and depression (Abramson et al., 2002), helplessness, and/or hopelessness (Kernis et al., 1989). Related to time effects, there is a significant body of research that examines the effects of repeated failure on a person’s achievement, including decreased self-worth (Vohs and Heatherton, 2001; Alicke and Sedikides, 2011), loss of self-esteem (Vohs and Heatherton, 2004), avoidance motivation, and eventually withdrawal (Oertig et al., 2013), and failure to self-regulate (Koch et al., 2008). Item position effects have the potential to trigger those devastating mechanisms because, if items appear to become progressively more difficult as the test progresses, the imminent threat to the self (Suls et al., 2002) may result in low self-esteem (Heatherton and Vohs, 2000) and failure to self-regulate (Suls et al., 2002; van Dellen et al., 2011). So, from the perspective of the participant, item positions can play a substantial impact on both achievement (Bulut, 2015; Bulut et al., 2016, 2017) as well as perceptions of success and failure (Hughes and Beer, 2013; Bulut et al., 2017).

The image that was been painted is made even more complicated by the influence that gender plays (Vohs and Heatherton, 2003; Krendl et al., 2008). Although we failed to locate a single study examining item position effects, Balart and Oosterveen (2019) reported that females sustained their performance in math and science longer compared to males, thus closing the gap on these domains, although maintaining higher achievement in verbal tasks. Thus, these findings examining test-length suggest the presence of gender differences favoring females compared to males. These favorable results in females testing performance have been attributed to females being more disciplined (Duckworth and Seligman, 2006), agreeable (Costa et al., 2001), more conscientious (Schmitt et al., 2008), and engaging more elaborate planning strategies such as self-correction and verification prior to completing the test (Naglieri, 1999).

Furthermore, item position effects may, from the perspective of the test, represent a significant threat to the internal validity of the measure and the item properties. For instance, having variable item difficulty estimates for a given item as a function of its position compromises an item’s validity. This is because item difficulty estimates are influenced by the item’s position in the test. Participation in later items by a person could be indicative of aberrant responding, which, once more, invalidates quality responding by that person (by lowering the person’s reliability). As a result, determining strategies to deal with item location effects and conducting tests to determine whether or not such effects are present are important tasks for ensuring the validity of measurement. The goal of the present study was to evaluate the roles of item position in terms of item difficulty levels in the assessment of aptitude in a teacher licensure test in Saudi Arabia.

## 2. Methods

### 2.1. Participants and procedures

Amongst 41,698 teachers who took on the Teacher General Licensure Test (TGLT) the present report engaged a random sample of 5,000 participants so that both population parameters will be preserved (due to randomization) and to avoid Type-I errors due to the large amounts of power associated with a sample of 40,000 participants. that is large enough but not excessive so that every minuscule effect would be deemed significant. Participants had full data across the measure. There were 2,225 males and 2,775 females and had a mean age of 32.5 years (SD = 4.28 ranging between 22 and 52 years of age). Specifically for gender, the mean age of males was 32 years and 6 months (SD = 4.85 ranging between 22 and 53 years); the mean age of females was 32 years and 6 months (SD = 3.75, ranging between 22 and 49 years). The mean number of teaching experience was 10.6 years for males (SD = 4.84) and 10.5 years for females (SD = 3.74). All were professional teachers who took the TGLT for certification purposes under the auspices of the Education and Training Evaluation Commission (ETEC), and the Ministry of Education at the Kingdom of Saudi Arabia. ETEC follows the declaration of Helsinki guidelines for the ethical treatment of participants in research involving human subjects.

### 2.2. Measure and procedures

The TGLT was designed to meet national and international standards in the areas of professional values and responsibilities, professional knowledge, and professional practice. Professional educational standards for teachers consist of the general educational part, which is shared with all teachers of other disciplines and is measured by the *Teacher General Licensure Test (GTLT)*, which is one of the large-scale assessments that is designed and developed by Qiyas Experts at the Education & Training Evaluation Commission (ETEC). This test aims to measure the content of professional standards for teachers, determine the level of proficiency required following the professional licensing regulations for teachers, and determine the strengths and the need for development in the dimensions covered by the standards. The three specific domains under evaluation are (a), *professional values and responsibilities* (b), *professional knowledge*, and (c) *professional practice.* The total number of items was 75 and were distributed across the following nine specific domains: (a) *Commitment to moderate Islamic values, professional ethics, and the promotion of national identity*, (b) *Continuing professional development*, (c) *Professional interaction with educators and society*, (d) *Familiarity with quantitative and linguistics skills*, (e) *Knowledge of the learner and how one learns*, (f) *Knowledge of general teaching methods*, (g) *Teaching planning and implementation*, (h) *Create interactive and supportive learning environments for the learner*, and, (i) *Evaluation.*

Supplementary Table 1 presents the methodology involved in the measurement of teachers’ professional skills and competencies via the TGLT. The instrument was administered using four different forms in which items were presented in various orders. For example, as shown in the Supplementary Table, item 1 showed up in positions 1, 23, 45, and 67. Blocks of items moved consistently across forms so that they would appear early, toward the middle of the test, or later on in the test.

### 2.3. Data analyses

**FIGURE 1:**
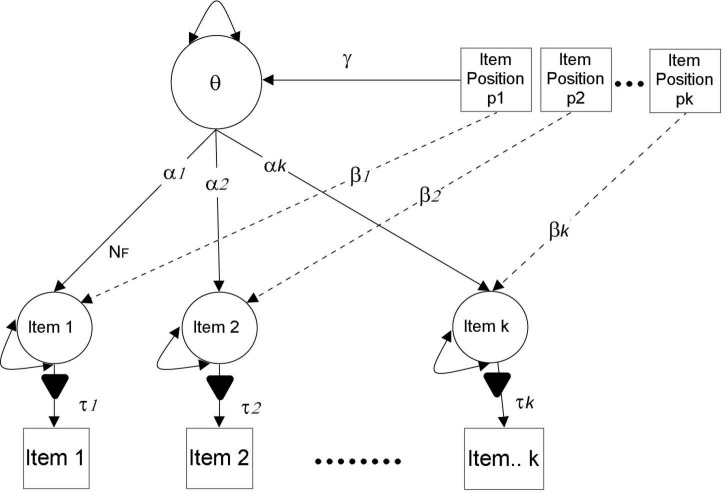
Modeling item position effects using MIMIC model in SEM framework.

In order to provide answers to the research questions posed in this study, the authors relied primarily on three different analytic approaches. The first two questions concerned determining whether or not there was gender-specific variation in measurement, with the end goal of determining whether or not there was gender-specific variation in the function that item position plays. To determine measurement invariance, tests of increased item parameter constraints were utilized, including the exact protocol of configural, metric, and scalar invariance (Vandenberg and Lance, 2000; Cheung and Rensvold, 2002; Millsap, 2011). In the event that this procedure is unsuccessful, we will opt for partial measurement invariance. After “satisfying” measurement, invariance, full or partial, the Multiple Indicators Multiple Causes (MIMIC) model will be engaged to examine the impacts of item position as well as the difference between those effects based on gender using the protocol developed by Bulut et al. (2017). Using these recommendations, the item slopes are predicted by a set of covariates called p1-pk, which indicate item positions (see Figure 1). In the current investigation, a multi-group MIMIC model was utilized so that an analysis of the similarity or dissimilarity of the effects of item position could be carried out across gender. All analyses were conducted using Mplus 8.10 (Muthén and Muthén, 1998–2017) and using a Maximum Likelihood Estimator using Robust standard errors (MLR).

## 3. Results

### 3.1. Factorial validity of the TGLT and measurement invariance across gender

A CFA model was applied to ensure that the 9-factor conceptualization fit the data well. After using the full instrument, although the fit was acceptable, several items failed to contribute significantly to their respective factors as based on values of their factor loadings. Consequently, items with negative or slopes close to zero were deleted in subsequent iterations with the final instrument comprised of nine domains and 63 items. Model fit was adequate [CFI = 0.911, TLI = 0.906, RMSEA = 0.018, RMSEA_95% C.I.s_ = 0.018–0.019, SRMR = 0.039]. The chi-square test was significant as expected due to modeling exact fit and the large sample size as miniscule deviations between observed and expected covariance matrices would be deemed large. Subsequently, the 9-factor correlated structure comprised the optimal structure for the GTLT. Item level estimates (standardized factor loadings and item thresholds) are shown in Table 1.

**TABLE 1:** Factor loadings and item thresholds for nine-factor correlated confirmatory factor analysis model of the GTLT.

Item no.	Factor loading	Loading S.E.	Item threshold	Item threshold S.E.
**Commitment to moderate Islamic values, professional ethics, and the promotion of national identity (F1)**				
1	0.348	0.025	−0.883	0.020
2	0.317	0.022	0.040	0.018
3	0.296	0.024	−0.711	0.019
4	0.233	0.024	−1.031	0.022
5	0.331	0.022	−0.648	0.019
6	0.206	0.021	0.320	0.018
**Continuing professional development (F2)**				
7	0.378	0.027	−1.173	0.023
8	0.233	0.022	−0.083	0.018
9	0.229	0.021	−0.013	0.018
10	0.491	0.027	−0.120	0.018
**Professional interaction with educators and society (F3)**				
11	0.454	0.020	−0.430	0.018
12	0.524	0.021	−0.025	0.018
13	0.054	0.020	−0.267	0.018
14	0.358	0.020	−0.380	0.018
15	0.231	0.020	−0.069	0.018
**Familiarity with quantitative and linguistics skills (F4)**				
16	0.216	0.024	0.998	0.021
17	0.126	0.020	−0.250	0.018
18	0.298	0.020	−0.087	0.018
19	0.442	0.019	0.073	0.018
20	0.178	0.022	0.514	0.019
21	0.198	0.020	0.224	0.018
22	0.413	0.021	−0.912	0.021
23	0.195	0.021	−0.557	0.019
24	0.184	0.026	1.043	0.022
25	0.099	0.020	0.147	0.018
26	0.409	0.021	−0.776	0.020
27	0.319	0.028	1.174	0.023
28	0.080	0.020	0.045	0.018
**Knowledge of the learner and how he learns (F5)**				
29	0.252	0.019	−0.016	0.018
30	0.301	0.019	0.018	0.018
31	0.565	0.017	0.382	0.018
32	0.408	0.017	−0.250	0.018
33	0.503	0.018	0.278	0.018
34	0.658	0.016	0.333	0.018
35	0.316	0.023	−1.164	0.023
36	0.321	0.019	−0.026	0.018
37	0.386	0.018	−0.252	0.018
**Knowledge of general teaching methods (F6)**				
38	0.291	0.026	0.680	0.019
39	0.245	0.025	0.688	0.019
40	0.264	0.025	0.604	0.019
41	0.430	0.027	−0.305	0.018
**Teaching planning and implementation (F7)**				
42	0.225	0.019	−0.180	0.018
43	0.427	0.020	0.356	0.018
44	0.277	0.020	0.484	0.018
45	0.413	0.019	0.165	0.018
46	0.440	0.020	−0.676	0.019
47	0.229	0.022	−1.049	0.022
48	0.124	0.019	0.137	0.018
49	0.239	0.020	−0.578	0.019
**Create interactive and supportive learning environments for the learner (F8)**				
50	0.275	0.026	0.934	0.021
51	0.556	0.020	−1.081	0.022
52	0.574	0.018	0.115	0.018
53	0.409	0.023	0.838	0.020
54	0.373	0.023	0.848	0.020
55	0.323	0.019	−0.430	0.018
56	0.168	0.025	1.073	0.022
**Evaluation (F9)**				
57	0.559	0.017	0.055	0.018
58	0.058	0.022	0.527	0.019
59	0.464	0.019	0.257	0.018
60	0.389	0.018	−0.509	0.019
61	0.077	0.023	0.783	0.020
62	0.456	0.018	−0.084	0.018
63	0.455	0.018	0.033	0.018

Before evaluating item position effects, however, it was important to confirm that the instrument functioned in equivalent ways across gender. This was important because item position effects would be rendered meaningless if the functioning of a given item was different in males compared to females. Thus, the observed effect would confound item content with item position, rendering any conclusions on item position invalid. Consequently, we initially applied the classic protocol of measurement invariance using the three required levels of configural, metric, and scalar invariance. Results indicated that almost all (except for 4 comparisons of configural vs. metric invariance and 1 for metric vs. scalar that held) tests of measurement invariance were significant pointing to failure at confirming metric and scalar equivalence across males and females (see Supplementary Table 2), thus, we proceeded with tests of partial measurement invariance, after adjusting the observed *p*-values for family wise error using the Holm–Bonferroni’s sequential corrective procedure (Holm, 1979), confirmed using the Šidák (1967) correction estimated as 1 − 1(1 − *a*)^1/*m*^ with m being the number of tests conducted.

When testing for measurement invariance across gender, 39 out of the 126 statistical tests constraining item factor loadings and thresholds to be equivalent were statistically significant representing 30.9% of the total number of tests. Based on the work of Steenkamp and Baumgartner (1998) partial measurement invariance is justified when at least half of the estimated parameters are invariant. However, in order to adjust the observed *p*-values for the number of tests (*m* = 126; 63 factor loadings + 63 thresholds) the observed *p*-values were adjusted using the Holm–Bonferroni’s sequential correction, supplemented with a corrected level of significance as suggested by Šidák (1967). The latter level of significance was equal to 0.0004 being adjusted for a family of 126 tests. Using both the Holm–Bonferroni’s and the Sidak procedures, results indicated that none of the observed *p*-values exceeded the corrected level of significance. Thus, both item slopes and item thresholds were considered largely invariant and the initially observed significant effects that were likely due to the large sample size and the uncorrected for multiple tests level of significance.

### 3.2. Item position effects on the TGLT

Initially the MIMIC model was applied to test item position effects using aggregated data. Results indicated that there were initially 7 significant effects (out of 63) none of which survived the Holm–Bonferroni’s sequential corrective procedure or the Sidak procedure. The largest item position coefficient was 0.004 logit, reflecting a small effect size. Thus, the overall conclusion was that item position effects on the GTLT were literally non-existent.

Table 2 presents the results from item positions and their difference across gender. As shown in the table, after correcting the level of significance for the number of tests conducted, no comparison exceeded levels of significance using the Holm–Bonferroni’s corrective procedure. Using the Sidak corrective procedure, however, which was apparently less conservative several tests (30 out of 63) were significant, albeit with small effect sizes. Nevertheless, despite the small effect sizes, a trend was apparent due to gender; That is, in all but four significant effects (87%) across gender, the difference coefficients were negative suggesting that females were taking more time in responding to the items likely reflecting a more general trend of being careful, focused and concentrated during test taking.

**TABLE 2:** Differences on item position effects per domain of the GTLT across gender.

Item no.	Item position males	Item position females	Difference item position estimate	S.E.	Decision Holm–B*	Decision Sidak*
**Commitment to moderate Islamic values, professional ethics, and the promotion of national identity (F1)**						
1	0.002	0.001	0.001	0.002	n.s.	n.s.
2	0.001	0.000	0.000	0.002	n.s.	n.s.
3	−0.005	0.004	−0.009	0.002	n.s.	Sig.
4	0.000	0.004	−0.005	0.002	n.s.	n.s.
5	−0.001	0.002	−0.003	0.002	n.s.	n.s.
6	0.002	0.006	−0.004	0.001	n.s.	n.s.
**Continuing professional development (F2)**						
7	0.002	0.000	−0.002	0.002	n.s.	n.s.
8	0.003	−0.001	−0.004	0.001	n.s.	n.s.
9	0.006	0.000	−0.005	0.001	n.s.	Sig.
10	0.004	−0.003	−0.008	0.001	n.s.	Sig.
**Professional interaction with educators and society (F3)**						
11	0.007	−0.003	−0.011	0.001	n.s.	Sig.
12	0.005	−0.006	−0.011	0.001	n.s.	Sig.
13	0.003	0.002	−0.001	0.001	n.s.	n.s.
14	0.000	−0.005	−0.005	0.001	n.s.	Sig.
15	−0.003	0.001	0.004	0.001	n.s.	Sig.
**Familiarity with quantitative and linguistics skills (F4)**						
16	−0.003	0.000	−0.003	0.001	n.s.	n.s.
17	0.001	0.002	−0.001	0.001	n.s.	n.s.
18	−0.005	0.001	−0.006	0.001	n.s.	Sig.
19	−0.005	0.001	−0.006	0.001	n.s.	Sig.
20	0.002	−0.001	0.003	0.001	n.s.	n.s.
21	−0.002	0.003	−0.005	0.001	n.s.	Sig.
22	−0.005	0.002	−0.007	0.002	n.s.	n.s.
23	0.002	0.001	0.001	0.001	n.s.	n.s.
24	0.004	0.003	0.001	0.002	n.s.	n.s.
25	0.006	−0.006	0.012	0.001	n.s.	Sig.
26	−0.001	−0.002	0.001	0.002	n.s.	n.s.
27	0.004	−0.006	0.010	0.002	n.s.	Sig.
28	−0.004	0.002	−0.006	0.001	n.s.	Sig.
**Knowledge of the learner and how he learns (F5)**						
29	0.000	0.006	−0.005	0.001	n.s.	Sig.
30	−0.005	0.003	−0.009	0.001	n.s.	Sig.
31	−0.006	0.004	−0.010	0.001	n.s.	Sig.
32	−0.003	0.000	−0.004	0.001	n.s.	n.s.
33	−0.004	0.002	−0.006	0.001	n.s.	Sig.
34	−0.010	0.006	−0.016	0.002	n.s.	Sig.
35	−0.004	0.000	−0.004	0.002	n.s.	n.s.
36	−0.003	0.005	−0.008	0.001	n.s.	Sig.
37	−0.002	0.001	−0.002	0.001	n.s.	n.s.
**Knowledge of general teaching methods (F6)**						
38	−0.003	0.004	−0.007	0.001	n.s.	Sig.
39	0.000	0.002	−0.002	0.001	n.s.	n.s.
40	−0.007	0.001	−0.008	0.002	n.s.	Sig.
41	−0.003	0.004	−0.007	0.001	n.s.	Sig.
**Teaching planning and implementation (F7)**						
42	−0.001	0.003	−0.004	0.001	n.s.	n.s.
43	−0.006	0.001	−0.007	0.002	n.s.	Sig.
44	−0.007	0.003	−0.010	0.001	n.s.	Sig.
45	−0.001	0.003	−0.004	0.001	n.s.	n.s.
46	0.003	0.004	−0.001	0.002	n.s.	n.s.
47	−0.009	0.005	−0.014	0.002	n.s.	Sig.
48	0.002	0.003	−0.001	0.001	n.s.	n.s.
49	0.000	0.001	−0.001	0.001	n.s.	n.s.
**Create interactive and supportive learning environments for the learner (F8)**						
50	−0.002	0.000	−0.001	0.001	n.s.	n.s.
51	−0.003	0.000	−0.003	0.002	n.s.	n.s.
52	−0.001	0.002	−0.003	0.001	n.s.	n.s.
53	−0.004	0.004	−0.007	0.001	n.s.	Sig.
54	−0.003	0.001	−0.004	0.001	n.s.	n.s.
55	−0.003	0.003	−0.006	0.001	n.s.	Sig.
56	0.000	−0.002	0.001	0.001	n.s.	n.s.
**Evaluation (F9)**						
57	−0.006	0.001	−0.007	0.001	n.s.	Sig.
58	0.000	0.001	−0.001	0.001	n.s.	n.s.
59	−0.003	0.004	−0.007	0.002	n.s.	Sig.
60	0.002	−0.004	0.006	0.002	n.s.	Sig.
61	0.001	0.000	0.001	0.002	n.s.	n.s.
62	−0.002	0.000	−0.002	0.001	n.s.	n.s.
63	−0.002	0.001	−0.007	0.001	n.s.	n.s.

n.s, non-significant effect. The different item position estimate reflects differences across gender on item difficulties in logits per one item position change. *Decision based on the comparison between the observed *p*-value and whether it was below the corrected level of significance using the Holm–Bonferroni’s sequential procedure or the Sidak procedure.

## 4. Discussion

The goal of the present study was to evaluate the roles of item position in terms of item difficulty levels in the assessment of aptitude in a teacher licensure test in Saudi Arabia. Several important findings emerged and are discussed next. The most important finding was that item position effects were present in a small percentage of the items (11%) before correction and zero after applying corrective procedures for multiple tests, suggesting that the extend of item position effects is very small to non-existent and likely localized to specific item content. Furthermore, in the instances when significant effects emerged, those were reflective of small effect sizes as judged by Cohen (1992).

A second important finding was that, in the case of significant effects, gender trends were evident with females spending more time on the items when they appeared later on a test compared to males. This was a finding that had crucial implications for future research although we can only speculate on whether females have enhanced attention to detail, motivation, concentration, and focus when confronted with the same topic in later phases. This finding is in agreement with the study by Balart and Oosterveen (2019), who revealed that in cognitively demanding activities, females exhibited a greater capacity to maintain their performance compared to males. Their findings led them to conclude that “female students would make greater use of the extra time on the test because of their capacity to sustain performance” (p. 2). The authors went on to indicate that three probable factors were likely responsible for the observed gender disparities. These potential sources were (a) females having higher non-cognitive skills, (b) females engaging more in test-taking methods, and (c) females exerting more effort while taking tests. If the disparities that were found are representative of motivational dispositions, then the data presented here contradict the conclusions of the study by Montrolio and Taberner (2021), which stated that male university students performed better when they were under time constraints, thus, making better use of available time.

### 4.1. Study limitations

The current study has several shortcomings. First, exact invariance was not achieved suggesting enhanced variability in several gender-specific item thresholds. Although these results diminished after applying corrective procedures for family wiser error, it is possible that some items may have been more difficulty in one gender. This difference in item difficulty levels may have, to some extent, influenced performance on the items and the exam. In addition, the random sample that was used still had an overwhelming level of power; hence, some of the findings may represent Type-I errors. Third, we could only account for gender differences in age and experience and could not rule out the hypothesis that gender differences existed in other important variables that potentially influence the outcomes of the present study (e.g., parental education, SES, motivation, etc.). Last, because we did not take any assessments of the participants’ motivation, affect, anxiety, or other personal dispositions, we are unable to draw inferences on whether and how these factors affected student performance during the test.

### 4.2. Implications of the present findings and future directions

In the present study, item position effects were not prevalent with the real data of the current national assessment. However, several ideas can be put forth to deal with the presence of serial item ordering. One strategy for dealing with item location effects is to use test equating, which involves adjusting for differences in difficulty across different versions or forms of a test. This helps to ensure that scores obtained from different versions of the test are comparable and that differences in scores are not solely due to differences in item difficulty. Another strategy is to randomize the order of items within the test. This can help to reduce the impact of item location effects on item difficulty estimates and on participants’ responses. By randomizing the order of items, the likelihood that participants will encounter items of similar difficulty in close proximity is reduced, which helps to minimize the impact of item location effects on participants’ performance. Additionally, it may be useful to conduct item analyses to identify potential item location effects. Item analyses can be used to examine the relationships between item difficulty estimates and item position, as well as to examine the relationships between participants’ responses to items and their position within the test. By identifying potential item location effects, researchers can take steps to minimize their impact on the measurement process and ensure the validity of the measure.

The continuation of this area of research can go in several interesting ways in the future. To begin, the location of an item on a test has a direct correlation to its total length. This makes obvious sense, given that longer examinations tend to be more taxing (Jensen et al., 2013). In light of the findings of the experiments, which showed that longer tests were associated with higher levels of perceived fatigue but also higher levels of performance, it is possible that an investigation into the interaction between the length of the test and levels of fatigue will provide insight into the causes of item position effects (Jensen et al., 2013). Second, because of the shift toward the use of electronic methods, such as computerized adaptive testing (CAT) and multistage testing (MST), it is essential to investigate the notion that item location effects are not a function of item exposure or test form effects. Last, investigations often ignore the possibility of concurrent operation of many item context effects by concentrating on only one sort of item context effects, such as item position effects or mode effects. Test fairness refers to the degree to which the test ensures procedural equality for individuals and subgroups of test-takers and the sufficiency of the representation of the construct in test materials and procedures (Johnson and Geisinger, 2022). In conclusion, investigating how item placements are linked to invalidity and aberrance in in-person response vectors is an exciting avenue of research to pursue (see Ferrando, 2007, 2009).

## Data availability statement

The raw data supporting the conclusions of this article will be made available by the authors, without undue reservation.

## Ethics statement

The studies involving human participants were reviewed and approved by the ETEC. The patients/participants provided their written informed consent to participate in this study.

## Author contributions

GS: conceptualization, data analyses, and write-up of draft manuscript. HH and FJ: provision of data, data analyses, write-up of manuscript, and approval of manuscript. All authors contributed to the article and approved the submitted version.
