# Supplementary material for: The item position effects in international examinations: the roles of gender
Source: Front Psychol. 2023 Aug 15;14:1220384. doi: 10.3389/fpsyg.2023.1220384 (PMC10465346; doi:10.3389/fpsyg.2023.1220384)
Supplement: Supplementary file 1 [file Data_Sheet_1.docx]

Supplementary Material

The Item Position Effects in International Examinations:

The Roles of Gender

Georgios Sideridis^1,2^*, Fathima Jaffari^3,4^, Hailah Hamed^5,4^

*** Correspondence:** Corresponding Author georgios.sideridis@childrens.harvard.edu

# Supplementary Tables

**Supplementary Table 1.** *Questions and item position for the first domain of the measure.*

| **Question Number of Zero Model** | **Domain Number** | **Model A** | **Model B** | **Model C** | **Model D** |
| --- | --- | --- | --- | --- | --- |
| 1 | 1 | 1 | 67 | 45 | 23 |
| 2 | 1 | 2 | 68 | 46 | 24 |
| 3 | 1 | 3 | 69 | 47 | 25 |
| 4 | 1 | 4 | 70 | 48 | 26 |
| 5 | 1 | 5 | 71 | 49 | 27 |
| 6 | 1 | 6 | 72 | 50 | 28 |

Supplementary Table 2. *Model Comparison for Testing Equivalence of Slopes (Metric Model) and Intercepts (Scalar Model) in Evaluating Differences Across Gender Using Exact Inferential Statistical Criteria.*

| **Model Tested** | **Chi-square Difference Test** | **D.F.** | **CFI** | **RMSEA** |
| --- | --- | --- | --- | --- |

| *Commitment to moderate Islamic values, professional ethics, and the promotion of national identity (F1)* |
| --- |

| F1. Configural vs. Metric | 4.516 | 6 | 0.946 | 0.012 |
| --- | --- | --- | --- | --- |
| F1. Metric vs. Scalar | 64.286*** | 6 | 0.603 | 0.030 |
| *Continuing professional development (CPD) (F2)* | | | | |
| F2. Configural vs. Metric | 10.369* | 4 | 0.944 | 0.018 |
| F2. Metric vs. Scalar | 86.092*** | 4 | 0.224 | 0.055 |
| *Professional interaction with educators and society (F3)* | | | | |
| F3. Configural vs. Metric | 5.433 | 6 | 0.962 | 0.014 |
| F3. Metric vs. Scalar | 195.277*** | 6 | 0.319 | 0.052 |
| *Familiarity with quantitative and linguistics skills (F4)* | | | | |
| F4. Configural vs. Metric | 22.268 | 13 | 0.870 | 0.015 |
| F4. Metric vs. Scalar | 277.830*** | 13 | 0.461 | 0.030 |
| *Knowledge of the learner and how he learns (F5)* | | | | |
| F5. Configural vs. Metric | 45.579*** | 11 | 0.920 | 0.026 |
| F5. Metric vs. Scalar | 463.950*** | 11 | 0.700 | 0.048 |
| *Knowledge of general teaching methods (F6)* | | | | |
| F6. Configural vs. Metric | 36.047*** | 6 | 0.688 | 0.027 |
| F6. Metric vs. Scalar | 1326.848*** | 6 | 0.000 | 0.048 |
| *Teaching planning and implementation (F7)* | | | | |
| F7. Configural vs. Metric | 28.231** | 11 | 0.886 | 0.017 |
| F7. Metric vs. Scalar | 255.262*** | 11 | 0.474 | 0.034 |
| *Create interactive and supportive learning environments for the learner (F8)* | | | | |
| F8. Configural vs. Metric | 49.854*** | 8 | 0.876 | 0.028 |
| F8. Metric vs. Scalar | 85.551*** | 8 | 0.774 | 0.035 |
| *Evaluation (F9)* | | | | |
| F9. Configural vs. Metric | 40.469*** | 10 | 0.916 | 0.022 |
| F9. Metric vs. Scalar | 129.594*** | 10 | 0.809 | 0.031 |

*Note:* **p*<0.05; ***p*<0.01; ****p*<0.001. D.F.=Degrees of Freedom; CFI=Comparative Fit Index; RMSEA=Root Mean Square Error of Approximation.
